# Editorial: 10 years of the JACMP

**DOI:** 10.1120/jacmp.v10i4.3195

**Published:** 2009-10-20

This issue marks the conclusion of 10 years of publication of the JACMP and, to note the occasion, I passed my editorial hat to Dr Peter Almond, the first Editor‐in‐Chief of the JACMP, and invited him to write a guest editorial for this issue of the journal. Before I temporarily relinquish the reins to Dr Almond, however, I want to make note of the fact that this issue of the JACMP is the largest yet published. Consequently, special thanks goes to the authors without whose contributions we would have no journal, the Associate Editors listed below, and the reviewers, listed in a Supplementary File.

Associate Editors (including Guest Associate Editors): Nzhde Agazaryan, Salahuddin Ahmad, John Antolak, Peter Balter, Sam Beddar, Pat Cadman, Marco Carlone, Nathan Childress, Geoff Clarke, Laurence Court, Larry DeWerd, Bill Erwin, John Gibbons, Michael Gossman, Rebecca Howell, Ed Jackson, Jennifer Johnson, Stephen Kry, Rajat Kudchadker, Moyed Miften, Robin Miller, Eduardo Moros, Firas Mourtada, Ben Nelms, John Pacyniak, Niko Papanikolaou, Matt Podgorsak, Jerimy Polf, Karl Prado, Jim Rodgers, John Rong, Isaac Rosen, Bill Salter, Mehrdad Sarfaraz, Jeff Shepard, Almon Shiu, Alf Siochi, Eric Siessinger, Tim Solberg, Jason Stafford, Frank Van Den Heuvel, Sastry Vedam, Lu Wang, Chuck Willis, Twyla Willoughby, Al Zacarias, and Ron Zhu.

You will hear from me again in February with Vol 11 Number 1.

George Starkschall, PhD

Editor‐in‐Chief

November 15, 2009

## Supporting information

Supplementary Material Files

