# Supplementary material for: Editorial: 10 years of the JACMP
Source: J Appl Clin Med Phys. 2009 Oct 20;10(4):1. doi: 10.1120/jacmp.v10i4.3195 (PMC5720578; doi:10.1120/jacmp.v10i4.3195)
Supplement: Supplementary file 1 — Supplementary Material Files [file ACM2-10-001-s001.pdf]

2009 Reviewer Record

Muhammad	Afghan
Vinod	Agrawal
Hilary	Akpati
Parham	Alaei
Mohamed	Alfifi
Imad	Ali
Will	Ansbacher
Louis	Archambault
Ben	Archer
Clement	Arsenault
Maria	Asparadakis
Mohammad	Bakhtiari
Lajos	Balogh
Stephen	Balter
Parminder	Basran
Daniel	Bassano
Peter	Biggs
Charles	Bloch
Stefan	Both
Scott	Brame
Tina	Briere
Debra	Brinkman
Dean	Broga
Paul	Brown
Terry	Button
Patrick	Cadman
Jean-Francois	Carrier
Zheng	Chang
Indrin	Chetty
Nathan	Childress
S-T	Chiu-Tsao
Weijun	Chow
James	Chow
Cynthia	Chuang
George	Ciangaru
Diana	Cody
Laurence	Court
Oana	Craciunescu
Gavin	Cranmer-Sargison
Wouter	Crijns
Adam	Cunha
Bruce	Curran
Ellen	Day
Tom	Depuydt
Peter	Dickof
Sonja	Dieterich
Nesrin	Dogan
Gary	Ezzell
Jessica	Fagerstrom
Kara	Ferachi

Marcus	Fischer
David	Followill
Rohini	George
Bruce	Gerbi
Lee	Gerig
John	Gibbons
Kent	Gifford
David	Gladstone
Devon	Godfrey
Steven	Goetsch
John	Gordon
Michael	Gossman
Edward	Graves
Mariana	Guerrero
Alonso	Gutierrez
Fred	Hacker
Alicia	Harris
Bret	Heintz
Peter	Hoban
Jan	Hrbacek
Sunyoung	Jang
Hongyu	Jiang
Jian-Yue	Jin
Kristofer	Kainz
Pantelis	Karaiskos
Brian	Kavanaugh
Joseph	Killoran
Jon	Kruse
Stephen	Kry
Rajat	Kudchadker
Lalith	Kumarasway
Alex	Kwan
Chao-Jen	Lai
Michale	Lamba
Katja	Langen
Guang	Li
Zuofeng	Li
Hua	Li
Yun	Liang
Lan	Lin
Dershan	Luo
Yulia	Lyatskaya
Eugene	Mah
Dennis	Mah
Harish	Malhotra
Rafael	Martin-Landrove
Martha	Matuszak
Osama	Mawlawi
Charles	Mayo
Marc	McKenzie
Helen	McNair
Ali	Meigooni

Robert	Meiler
Geetha	Menon
Michael	Mills
Takashi	Mizowaki
Rafael	Mockli
Yildrim	Mutaf
Sasa	Mutic
Daryl	Nazareth
Daniel	Neck
Malgorzata	Niedbala
Michelle	Nielsen
Paige	Nitsch
Kent	Ogden
Michael	Oliver
Orest	Ostapiak
Asa	Palm
Tinsu	Pan
Lech	Papiez
William	Parker
Brent	Parker
Kamen	Paskalev
Daniel	Pavord
Donald	Peck
Yong	Peng
Edward	Pennington
Dinko	Plenkovich
Darrell	Poole
Richard	Popple
Jean	Pouliot
Sharon	Qi
Dharanipathy	Rangaraj
Prema	Rassiah
Satyapal	Rathee
Larry	Reinstein
Susan	Richardson
Mark	Rivard
James	Robar
Yi	Rong
Chun	Ruan
Narayan	Sahoo
Laskhmi	Santanam
Paolo	Scalchi
Deborah	Schofield
Eric	Schreiber
Eduard	Schreibmann
Brad	Schuller
Rufus	Scrimber
Chengyu	Shi
Almon	Shiu
Alfredo	Siochi
Matt	Skinner
Stan	Skubic

Ron	Sloboda
James	So
William	Song
Ingrid	Spadinger
Spiridon	Spirou
Sotirios	Stathakis
Ned	Sternick
Brinda	Subramanian
Phillip	Taddei
Russell	Tarver
Steve	Tenn
Bruce	Thomadsen
Uwe	Titt
Ranjini	Tolakanahali
Philip	Tschou
Marcel	Van Herl
Kenneth	Vanek
Sastry	Vedam
Carlos	Velasco
Vaidehi	Venkatakrishnan
Maria	Vlachaki
Louis	Wagner
Thomas	Wagner
Daniela	Wagner
Robert	Wallace
Chuang	Wang
Zhiheng	Wang
Deming	Wang
Jian	Wang
Congjun	Wang
He	Wang
Yun	Wang
Jihong	Wang
Heather	Warkentin
Charles	Willis
Brian	Winey
Qiuwen	Wu
Tao	Wu
Ping	Xia
Hong	Xiang
Weijun	Xiong
Scott	Yakubian
Guanghua	Yan
Jinsong	Ye
Byong Yong	Yi
Lifeng	Yu
Omar	Zeidan
Hui	Zhao
